# Looking Forward to Ochsner's 81st Year

**DOI:** 10.31486/toj.22.5029

**Published:** 2022

**Authors:** Ronald G. Amedee

**Affiliations:** Director of Clinical School and Professor, The University of Queensland Medical School, Ochsner Clinical School, New Orleans, LA; Editor-in-Chief, *Ochsner Journal*

*Gratitude is a quality similar to electricity; it must be produced and discharged and used up in order to exist at all.*

*–William Faulkner*

The Winter 2022 issue of the *Ochsner Journal* contains four original research articles, one quality improvement project and one innovative program report, and seven case reports and clinical observations that serve as the principal elements for this edition. You will also definitely want to read the two editorials submitted by gifted faculty leaders in the continuum of medical education at Ochsner Health, Drs Suma Jain and Bobby Nossaman. Dr Jain offers a heartfelt reflection on our experiences with COVID during the past 2½ years. Dr Nossaman explains the origin of the Hawthorne effect, describes how it manifests in medicine, and provides additional data supporting an important research article from Ochsner's Department of Anesthesiology and Perioperative Medicine. That article, “Perioperative Efficiency of Sugammadex Following Laparoscopic Cholecystectomy in Clinical Practice,” is a bit of a smackdown of the generally accepted perception that sugammadex is associated with not-negligible time savings in the operating room and the postanesthesia care unit.

We have a couple of orthopedics-related research papers with noteworthy foci. A team from Luminis Health in Annapolis, Maryland, presents their findings about the effect of factors such as household income, marital status, education level, and stress on surgical outcomes in “Social Determinants of Health Influence Early Outcomes Following Lumbar Spine Surgery.” An Ochsner orthopedics team led by Dr George Chimento delved into the relationship between the incidence of infection after total joint arthroplasty and the time of year when the surgery was performed in “Seasonal Relationship of Prosthetic Joint Infection Following Primary Total Joint Arthroplasty in a Subtropical Climate: A Retrospective Cohort Study.”

Our colleagues in Australia propose a definition of frequent presenters to the emergency department (no current consensus exists on what constitutes a frequent presenter), as well as a new way to identify, track, and manage these patients in “Monthly Identification of High Frequency Emergency Presenters to Improve Care Delivery and Evaluation: A Unique Methodological Approach.” They maintain that examining data on a rolling monthly basis, rather than an annual basis, could help facilitate timely case review and care delivery.

Over in the United Kingdom, Burnett-Jones et al developed a quality improvement project to address incomplete compliance with National Institute for Health and Care Excellence guidelines. They report their strategy and results in “Intervention to Improve Compliance With National Guidelines on Venous Thromboembolism Chemoprophylaxis for Patients With Operatively Managed Ankle Fractures.”

Over in Baton Rouge, Louisiana, Ardoin et al took a page from the undergraduate medical education playbook and trialed a problem-based learning curriculum with internal medicine interns. Ardoin and her team developed four cases addressing common chief complaints encountered in the hospital, and small groups of learners worked through the cases on Friday afternoons—developing problem lists and differentials, and interpreting labs and imaging. “Integrating Problem-Based Learning Into an Internal Medicine Residency Curriculum” includes previews of two of their cases, as well as samples of the handouts. The authors are willing to share a complete set of materials on request.

A fascinating case from Ochsner's Departments of Neurology and Transplant, “Three-Dimensional Visualization With Virtual Reality Facilitates Complex Live Donor Renal Transplant,” details how cutting-edge virtual reality and 3-dimensional modeling tools were used to re-evaluate a kidney donor's anatomy. The selection committee initially denied the donor because of his kidney's vascular complexity, but the models provided essential detail to demonstrate that the left kidney was acceptable, and the donor was able to provide the organ for his brother.

Other case reports included in this issue are from clinical teams in California, Kansas, Nebraska/Texas, India, Pakistan, and Thailand and address a variety of medical scenarios: hyperemesis gravidarum, autosomal recessive osteopetrosis, spontaneous coronary artery dissection, solitary fibrous tumor, RAD-140 drug-induced liver injury, and *Burkholderia gladioli* pneumonia in a patient with COVID-19.

The publication of this final issue of 2022 occurs as we conclude Ochsner's 80th anniversary celebration. We have much to be thankful for as we enter our 81st year at Ochsner Health. The dawn of a new year is always a time to reflect on the accomplishments and struggles of the past one, while forwardly focusing our view on the opportunities that 2023 will certainly offer us. This institution is a grateful one and continuously recognizes the efforts of its many dedicated leaders, practitioners, staff, and support teams who make our success as a health care organization possible.

The *Ochsner Journal* is also grateful to its reviewers and its editorial and publication teams who make this publication possible. To the many authors who submit their work to us for consideration, please accept our sincerest thank you, as your manuscripts serve as the lifeblood of our *Journal.* Please continue submitting to the *Journal* and reading this body of work.

Happy Holidays to all.

